# Клинико-лабораторные особенности ревматоидного артрита у мужчин в зависимости от уровня тестостерона

**DOI:** 10.14341/probl13373

**Published:** 2023-11-02

**Authors:** Т. С. Паневин, Р. В. Роживанов, Е. Г. Зоткин, М. Е. Диатроптов, С. И. Глухова, Е. Ю. Самаркина

**Affiliations:** Научно-исследовательский институт ревматологии им. В.А. Насоновой; Дальневосточный государственный медицинский университет; Национальный медицинский исследовательский центр эндокринологии; Научно-исследовательский институт ревматологии им. В.А. Насоновой; Научно-исследовательский институт ревматологии им. В.А. Насоновой; Научно-исследовательский институт ревматологии им. В.А. Насоновой; Научно-исследовательский институт ревматологии им. В.А. Насоновой

**Keywords:** ревматоидный артрит, тестостерон, гипогонадизм, андрогенодефицит

## Abstract

**ОБОСНОВАНИЕ:**

ОБОСНОВАНИЕ. Предполагается, что наличие хронического иммуновоспалительного ревматического заболевания (ИВРЗ) может быть фактором, увеличивающим вероятность развития синдрома гипогонадизма, и наоборот — наличие некомпенсированного дефицита тестостерона может предрасполагать к большему риску развития или более тяжелому течению ИВРЗ.

**ЦЕЛЬ:**

ЦЕЛЬ. Изучить частоту гипогонадизма у мужчин с ревматоидным артритом (РА) и оценить его влияние на течение РА и сопутствующие заболевания.

**МАТЕРИАЛЫ И МЕТОДЫ:**

МАТЕРИАЛЫ И МЕТОДЫ. В одномоментное сплошное исследование включено 170 мужчин с РА, находившихся на стационарном лечении в ФГБНУ НИИР им. В.А. Насоновой. Пациентам выполнено определение уровня общего тестостерона с последующим разделением на подгруппы с нормальным (>12 нмоль/л) и сниженным его уровнем. Проведено межгрупповое сравнение по основным показателям, используемым в клинической ревматологической практике для оценки стадии, активности и других медико-демографических характеристик РА, а также по состоянию пуринового и углеводного обменов. Был выполнен корреляционный анализ между уровнем общего тестостерона и некоторыми клинико-лабораторными показателями.

**РЕЗУЛЬТАТЫ:**

РЕЗУЛЬТАТЫ. Частота выявленного дефицита тестостерона в исследуемой группе составила 24,1%. Были отмечены значимые корреляционные связи между уровнем общего тестостерона и индексом массы тела (ИМТ) (r=-0,29), уровнем мочевой кислоты крови (r=-0,19) и С-реактивного белка (r=-0,18). Пациенты с гипогонадизмом по сравнению с группой с нормальным уровнем тестостерона характеризовались более высокими индексом массы тела (29,3±5,6 vs 26,3±4,0 кг/м2; p<0,001), уровнем глюкозы (6,95±7,85 ммоль/л vs 5,42±1,13 ммоль/л; p=0,034) и мочевой кислоты (354,6±110,7 vs 317,5±84,8 мкмоль/л; p=0,03) крови. Кроме того, пациенты с гипогонадизмом чаще страдали ожирением (41,6% vs 15,7%; p=0,001) и сахарным диабетом (21,6% vs 10,2%; p=0,075) без статистически значимой разницы, а также имели более высокий показатель СОЭ (46,5±42,2 vs 31,0±30,9 мм/ч; p=0,012). Отмечена более частая встречаемость анемии при гипогонадизме (32,4% vs 16,7%; p=0,041).

**ЗАКЛЮЧЕНИЕ:**

ЗАКЛЮЧЕНИЕ. Уровень тестостерона и наличие гипогонадизма не были связаны со стадией и активностью РА, однако дефицит тестостерона сопровождался более частым развитием избыточной массы тела и ожирения, и ухудшением пуринового и углеводного обменов.

## Abstract (en)

**BACKGROUND:**

BACKGROUND: It has been suggested that the presence of chronic immunoinflammatory rheumatic disease (CIRD) may be a factor that increases the likelihood of developing hypogonadism syndrome, and conversely, the presence of uncompensated testosterone deficiency may predispose to a greater risk of developing or more severe course of ICRD.

**AIM:**

AIM: To study the incidence of hypogonadism in men with rheumatoid arthritis (RA) and evaluate its impact on the course of RA and concomitant diseases.

**MATERIALS AND METHODS:**

MATERIALS AND METHODS: A one-time continuous study included 170 men with RA who were undergoing inpatient treatment at the Federal State Budgetary Institution NIIR named after. V.A. Nasonova. Patients were assessed for total testosterone levels and subsequently divided into subgroups with normal (>12 nmol/l) and reduced levels. An intergroup comparison was carried out on the main indicators used in clinical rheumatological practice to assess the stage, activity and other medical and demographic characteristics of RA, as well as the state of purine and carbohydrate metabolism. A correlation analysis was performed between the level of total testosterone and some clinical and laboratory parameters.

**RESULTS:**

RESULTS: The frequency of detected testosterone deficiency in the study group was 24.1%. Significant correlations were noted between the level of total testosterone and body mass index (r=-0.29), the level of blood uric acid (r=-0.19) and C-reactive protein (r=-0.18). Patients with hypogonadism compared to the group with normal testosterone levels were characterized by higher body mass index (29.3±5.6 vs 26.3±4.0 kg/m2; p<0.001), glucose levels (6.95±7 .85 mmol/l vs 5.42±1.13 mmol/l; p=0.034) and uric acid (354.6±110.7 vs 317.5±84.8 µmol/l; p=0.03) blood. In addition, patients with hypogonadism were more likely to suffer from obesity (41.6% vs 15.7%; p=0.001) and diabetes mellitus (21.6% vs 10.2%; p=0.075) without a statistically significant difference, and also had higher ESR (46.5±42.2 vs 31.0±30.9 mm/h; p=0.012). A more frequent occurrence of anemia was noted in hypogonadism (32.4% vs 16.7%; p=0.041).

**CONCLUSION:**

CONCLUSION: Testosterone levels and the presence of hypogonadism were not associated with the stage and activity of RA, however, testosterone deficiency was accompanied by a more frequent development of overweight and obesity, and a deterioration in purine and carbohydrate metabolism.

## Обоснование

Ревматоидный артрит (РА) является наиболее распространенным иммуновоспалительным ревматическим заболеванием (ИВРЗ), встречающимся у 0,5–1% населения и приводящим к серьезным социально-экономическим последствиям и сокращению продолжительности жизни пациентов [1]. За последнее десятилетие были зарегистрированы новые генно-инженерные биологические препараты, а также ингибиторы Янус-киназ, применение которых позволяет достичь низкой активности или ремиссии у большинства пациентов [2]. Однако даже при соблюдении текущих рекомендаций существует группа пациентов, у которых не удается достичь клинической ремиссии или низкой активности заболевания [3].

Гипогонадизм у мужчин — это синдром, связанный с низким уровнем тестостерона, а также нечувствительностью рецепторного аппарата к нему и его метаболитам, который может оказывать негативное воздействие на множество органов и систем, ухудшая качество жизни и жизненный прогноз. Распространенность гипогонадизма в мужской популяции варьирует от 5% у лиц без наличия серьезных хронических заболеваний до 30% при наличии сахарного диабета 2 типа и ожирения [4].

Предполагается, что наличие хронического ИВРЗ может быть фактором, увеличивающим вероятность развития синдрома гипогонадизма, и наоборот — наличие некомпенсированного дефицита тестостерона может предрасполагать к большему риску развития или более тяжелому течению ИВРЗ [5].

## ЦЕЛЬ ИССЛЕДОВАНИЯ

Изучить частоту гипогонадизма у мужчин с ревматоидным артритом и оценить влияние гипогонадизма на течение РА и сопутствующих заболеваний.

## МАТЕРИАЛЫ И МЕТОДЫ

## Место и время проведения исследования

Место проведения. ФГБНУ НИИР им. В.А. Насоновой

Время исследования. Январь 2022 — июнь 2023 гг.

## Изучаемые популяции

Критерии включения: мужской пол, установленный диагноз ревматоидного артрита согласно соответствующим классификационным критериям РА ACR/EULAR 2010 г.

Критерии исключения: наличие анамнеза применения андроген-депривационной терапии по поводу злокачественных новообразований, наличие онкологических заболеваний, сопровождающихся гиперэстрогенией, а также прием препаратов тестостерона или его синтетических производных и стимуляторов синтеза эндогенного тестостерона.

## Способ формирования выборки из изучаемой популяции (или нескольких выборок из нескольких изучаемых популяций)

Сплошной (пациенты, поступавшие на стационарное лечение).

## Дизайн исследования

Одноцентровое одномоментное исследование.

## Методы

Критерии включения и исключения определялись на основании анамнестических данных. У всех пациентов оценивались стандартные клинические и лабораторные показатели, определяемые в рамках лечения в ревматологическом стационаре.

Определение уровня тестостерона проводилось на анализаторе Cobas e411 с помощью наборов Elecsys Testosterone II Elecsys and cobas e analyzers/TESTO II (Roche Diagnostics GmbH). Гипогонадизм диагностировался при выявленном уровне общего тестостерона крови ≤12,0 нмоль/л. В последующем исследуемые были разделены на подгруппы в зависимости от наличия гипогонадизма, проведен межгрупповой сравнительный анализ.

## Статистический анализ

Для статистической обработки результатов использовались методы параметрической и непараметрической статистики программы Statistica 12.5 (StatSoft Inc., США). В зависимости от характера распределения результаты представлены в виде среднего значения и стандартного отклонения или медианы с интерквартильным размахом (Ме [ 25-й; 75-й процентили]). Для межгруппового сравнения количественных показателей в зависимости от нормальности распределения использовался t-критерий Стьюдента или U-критерий Манна-Уитни. Для сравнения качественных признаков использовался χ-квадрат Пирсона. Для изучения взаимосвязи уровня тестостерона и других количественных показателей проводился корреляционный анализ по Спирмену. Различия считались статистически значимыми при р<0,05.

## Этическая экспертиза

Исследование одобрено локальным этическим комитетом при ФГБНУ НИИР им.  В.А. Насоновой, протокол №2 от 27.01.2022 г., дата подписания: 27.01.2022 г.

## Результаты

В исследование было включено 170 пациентов. Средний возраст составил 54,8±12,3 года. Большинство пациентов имели развернутую или позднюю стадии РА, были серопозитивны по ревматоидному фактору и антителам к циклическим цитрулиннированным пептидам, имели умеренную или высокую активность, вторую/третью рентгенологическую стадию. Как минимум одно внесуставное проявление РА отмечалось почти у каждого второго пациента. Помимо базисной противовоспалительной терапии, каждый второй пациент на момент включения в исследование принимал метилпреднизолон, а каждый третий — НПВП. Большинство пациентов имели нормальную или избыточную массу тела, в то время как ожирение встречалось лишь в 19% случаев, а сахарный диабет — в 11,2%. Подробная клиническая характеристика пациентов представлена в табл. 1.

**Table:** Таблица 1. Подробная клиническая характеристика пациентов, включенных в исследование Примечание: РА — ревматоидный артрит; РФ — ревматоидный фактор; АЦЦП — антитела к циклическим цитруллинированным пептидам; АНФ — антинуклеарный фактор; DAS — disease activity score; ЧБС — число болезненных суставов; ЧПС — число припухших суставов; НПВП — нестероидные противовоспалительные препараты; ГИБТ — генно-инженерная биологическая терапия; СОЭ — скорость оседания эритроцитов; ИМТ — индекс массы тела; СКФ — скорость клубочковой фильтрации.

Показатель	Среднее±SD, медиана [ Q25; Q75], %
Тестостерон (нмоль/л)	17,0±7,4
Уровень тестостерона ≤12,0 нмоль/л	24,1%
Возраст (лет)	54,8±12,3
Стаж РА (лет)	6,0 [ 2,5; 10,0]
Стадия РА Очень ранняя Ранняя Развернутая Поздняя	0,6% 6,5% 64,7% 28,2%
Серопозитивность по РФ	84,0%
РФ (ед/л)	149 [ 36; 440]
Серопозитивность по АЦЦП	77,5%
АЦЦП (ед/л)	67,1 [ 10,3; 200,0]
Серопозитивность по АНФ	70,3%
Активность РА Ремиссия Низкая Умеренная Высокая	0,6% 1,8% 42,9% 54,7%
Индекс активности DAS28	5,2±1,1
ЧБС	8,8±5,6
ЧПС	5,2±4,4
Эрозивная форма	82,4%
Рентгенологическая стадия 1 2 3 4	4,1 53,5 28,8 13,5
Внесуставные проявления Ревматоидные узелки Синдром Шегрена Интерстициальное поражение легких Другое	44,7% 20,4% 14,1% 12,4% 5,3%
Класс функциональной недостаточности I II III IV	1,2% 78,8% 17,6% 2,4%
Получаемое лечение на текущий момент Метотрексат Лефлуномид Сульфасалазин Гидроксихлорохин Метилпреднизолон НПВП	45,9% 27,6% 12,9% 11,2% 51,8% 34,7%
Предшествующая ГИБТ	30,6%
Локальное введение ГК в анамнезе	27,6%
Средняя доза метотрексата (мг/нед)	18,5±5,2
Средняя доза метилпреднизолона (мг/сут)	6,0±3,1
Гемоглобин (г/л)	141,9±16,0
Анемия (Hb<130 г/л)	20,0%
Гематокрит (%)	45,3±30,8
СОЭ (мм/ч)	34,8±34,5
С-реактивный белок (мг/л)	10,1 [ 3,9;31,8]
ИМТ (кг/м²)	27,0±4,6
Ожирение ≥1 степени	19,0%
Глюкоза (ммоль/л)	5,8±4,0
Сахарный диабет	11,2%
Креатинин (мкмоль/л)	78,7±20,2
СКФ (CKD-EPI, мл/мин/1,73)	103,3±20,6
Мочевая кислота (мкмоль/л)	326,6±92,9
Артериальная гипертензия	54,1%
Ишемическая болезнь сердца	7,6%
Остеопороз	10,6%

Частота дефицита тестостерона в исследуемой группе составила 24,1%. Выявлены следующие значимые связи с уровнем общего тестостерона: слабая отрицательная связь с ИМТ (r=-0,29), уровнем мочевой кислоты крови (r=-0,19), уровнем С-реактивного белка (r=-0,18). По остальным количественным показателям, имеющим клиническое значение, статистически значимой корреляции не отмечалось (табл. 2).

**Table:** Таблица 2. Корреляция основных количественных показателей с уровнем тестостерона * значимая корреляция. Примечание: РА — ревматоидный артрит; РФ — ревматоидный фактор; АЦЦП — антитела к циклическим цитруллинированным пептидам; АНФ — антинуклеарный фактор; DAS — disease activity score; ЧБС — число болезненных суставов; ЧПС — число припухших суставов; СОЭ — скорость оседания эритроцитов; ИМТ — индекс массы тела; СКФ — скорость клубочковой фильтрации.

Возраст	-0,0814 (p=0,29)
Стаж РА (лет)	0,0845 (p=0,27)
DAS28Срб	-0,0309 (p=0,69)
ЧБС	0,0483 (p=0,53)
ЧПС	-0,0271 (p=0,72)
ИМТ	-0,2913 (p<0,001)*
Гемоглобин	0,1394 (p=0,07)
Гематокрит	0,1130 (p=0,14)
СОЭ	-0,0992 (p=0,2)
Глюкоза	-0,1366 (p=0,077)
Креатинин	-0,0762 (p=0,32)
СКФ (CKD-EPI)	0,0509 (p=0,51)
Мочевая кислота	-0,1894 (p=0,017)*
С-реактивный белок	-0,1842 (p=0,017)*
АНФ (титр)	-0,1271 (p=0,17)
РФ	0,2243 (p=0,008)*
АЦЦП	0,0477 (p=0,59)

При сравнении количественных показателей в подгруппах пациентов с нормальным и сниженным (≤12,0 нмоль/л) уровнем тестостерона (табл. 3) при сопоставимом возрасте гипогонадизм был ассоциирован со значимо более высоким средним показателем ИМТ (29,3±5,6 vs 26,3±4,0 кг/м²; p<0,001). Также отмечены более высокие показатели глюкозы крови натощак (6,95±7,85 ммоль/л vs 5,42±1,13 ммоль/л; p=0,034) и мочевой кислоты (354,6±110,7 vs 317,5±84,8 мкмоль/л; p=0,03). Кроме того, пациенты с гипогонадизмом чаще страдали ожирением (41,6% vs 15,7%; p=0,001) и незначимо чаще — сахарным диабетом (21,6% vs 10,2%; p=0,075). Распространенность артериальной гипертензии, ишемической болезни сердца и остеопороза в обеих группах не различалась.

**Table:** Таблица 3. Межгрупповое сравнение основных количественных показателей при нормальном и сниженном уровне тестостерона *значимые межгрупповые различия согласно t-критерию Стьюдента. DAS — disease activity score; ЧБС — число болезненных суставов; ЧПС — число припухших суставов; СОЭ — скорость оседания эритроцитов; ИМТ — индекс массы тела; СКФ — скорость клубочковой фильтрации.

Возраст (лет)	54,5±12,7	55,9±11,2	0,5
DAS28Срб	5,1±1,1	5,3±1,1	0,4
ЧБС	9,0±5,6	8,3±5,7	0,5
ЧПС	5,0±4,3	5,6±4,7	0,4
ИМТ (кг/м²)	26,3±4,0	29,3±5,6	0,0003*
Гемоглобин (г/л)	143,3±15,2	137,3±17,6	0,037*
Гематокрит	46,6±34,9	41,3±7,6	0,3
СОЭ (мм/ч)	31,0±30,9	46,5±42,2	0,012*
Глюкоза (ммоль/л)	5,4±1,1	6,95±7,9	0,034*
Креатинин (мкмоль/л)	77,8±17,6	81,4±26,7	0,3
СКФ (CKD-EPI, мл/мин/1,73)	104±20,6	100±20,5	0,3
Мочевая кислота (мкмоль/л)	317,5±84,8	354,6±110,7	0,03*

Помимо этого, показатели общего анализа крови также различались: при сниженном уровне тестостерона выявлена более высокая СОЭ (46,5±42,2 vs 31,0±30,9 мм/ч; p=0,012), а также более частая встречаемость анемии (уровень гемоглобина менее 130 г/л): 32,4% vs 16,7%; p=0,041.

Не было выявлено значимых межгрупповых различий по возрасту, основному индексу, отражающему активность РА (DAS28Срб), числу болезненных и припухших суставов, уровням ревматоидного фактора (РФ) и антител к циклическим цитрулиннирующим пептидам (АЦЦП), используемым в настоящее время дозам метотрексата и метилпреднизолона, концентрации С-реактивного белка.

## Обсуждение

Взаимосвязь между уровнем андрогенов и некоторыми ревматическими заболеваниями отмечена в исследованиях. Так, гипогонадизм у мужчин, не получавших тестостерон-заместительную терапию, ассоциировался с повышенным риском развития как РА (ОР 1,31; 95% ДИ 1,22–1,44), так и системной красной волчанки (ОР 1,58; 95% ДИ 1,28–1,94) [6], в то время как прием андроген-депривационной терапии также был связан с увеличением риска развития РА [7].

Данные результаты могут быть обусловлены возможным влиянием половых гормонов на иммунную систему, и наоборот. В патогенезе ряда ревматических заболеваний важную роль играет повышение синтеза провоспалительных цитокинов, в то время как при гипогонадизме может увеличиваться количество жировой ткани, макрофаги и адипоциты которой также способны продуцировать провоспалительные цитокины. С другой стороны, такие провоспалительные цитокины, как интерлейкин-1β (ИЛ-1β) и фактор некроза опухоли-α (ФНО-α), способны снижать выработку тестостерона, оказывая влияние на ось гипоталамус-гипофиз-яичко, а также непосредственно в клетках Лейдига за счет угнетения активности ферментов стероидогенеза и активации ароматазы. Кроме того, снижение уровня тестостерона сопровождается активацией врожденного иммунитета, в частности пути аутовоспаления NF-κB [5]. Напротив, терапия тестостероном оказывает ингибирующий эффект на продукцию провоспалительных цитокинов, угнетая экспрессию молекул адгезии и ИЛ-1β, ИЛ-6 и ФНО-α в различных типах клеток [8].

Исследования андрогенного статуса у мужчин с РА представлены преимущественно ретроспективными исследованиями с небольшим объемом выборок. В исследовании Прокаевой Т.Б., проведенном в начале 1990-х гг., среди 56 мужчин с РА были отмечены значимо более низкие уровни общего и свободного тестостерона при более высоких значениях ЛГ и ФСГ по сравнению с группой контроля [9].

В шведском исследовании по типу «случай-контроль», проведенном на выборке 104 мужчин с ранним РА в возрасте от 30 до 69 лет (медиана 59 лет), выявлена отрицательная корреляция между уровнем тестостерона и активностью РА [10]. Гипогонадизм был выявлен у 31,7% пациентов с РА в сравнении с 7,1% группы контроля. Также отмечено, что пациенты старше 50 лет имели низкий уровень лютеинизирующего гормона (ЛГ) (4,3±3,3 Ед/л vs 6,2±2,1 Ед/л, p=0,001) при низком уровне тестостерона, что может указывать на центральный генез андрогенодефицита.

В исследовании Кондрашова А.А. и соавт. среди 59 пациентов с РА в возрасте 50–70 лет снижение уровня общего тестостерона менее 12 нмоль/л выявлено у 13,6%, в то время как снижение уровня свободного тестостерона менее 225 нмоль/л — у 49,2% [11]. Уровень общего тестостерона отрицательно коррелировал с ИМТ (r=-0,52, р<0,001).

В нашем исследовании не было выявлено значимых различий в отношении активности, рентгенологической стадии, внесуставных проявлений РА, а также класса функциональной недостаточности в подгруппах с нормальным и сниженным уровнем тестостерона, хотя пациенты с андрогенодефицитом имели более высокие показатели СОЭ. Вместе с тем была обнаружена слабая отрицательная корреляционная связь между уровнем тестостерона и содержанием С-реактивного белка. Отсутствие различий по основным показателям активности (индекса DAS28, при расчете которого используются показатели СОЭ и С-реактивного белка) можно объяснить как недостаточным влиянием тестостерона на клинические проявления РА, так и особенностями выборки, которая была представлена больными в развернутой и поздней стадии заболевания (средняя продолжительность РА составляла 6 лет).

Выявленные различия в показателях ИМТ более частой встречаемости ожирения, а также в более высоком уровне глюкозы и мочевой кислоты при гипогонадизме у пациентов с РА открывают дискуссию о причинах данных изменений. В ряде исследований показана взаимосвязь между дефицитом тестостерона и развитием абдоминального ожирения, сахарного диабета 2 типа, артериальной гипертензии, инсулинорезистентности и дислипидемии [12], в то время как применение тестостерон-заместительной терапии может способствовать снижению массы тела и окружности талии у пациентов с СД2 [13]. С другой стороны, само по себе ожирение является фактором риска снижения уровня тестостерона. Следует отметить, что в нашем исследовании большая часть пациентов не страдала ожирением, хотя и имела избыточную массу тела.

## Ограничения исследования

Ограничением исследования является отсутствие клинической оценки гипогонадизма. Однако наиболее часто используемый для скрининга симптомов опросник AMS по результатам предшествующих исследований показал низкую специфичность, что, вероятно, связано с наличием хронического воспалительного поражения суставов, которое может влиять как на сексуальные аспекты, так и на результаты соматических и психологических вопросов в AMS. Так, в ранее упомянутом исследовании [11] при использовании опросника AMS среди пациентов с РА «андрогенодефицит» был выявлен в 87,5% случаев. При проведении корреляционного анализа между данными опросника AMS и активностью заболевания по DAS28 была выявлена умеренная прямая корреляция (r=0,324, р=0,001). При сравнении отдельных компонентов опросника с активностью заболевания имелась умеренная прямая корреляция с соматическими симптомами (r=0,451, р<0,001) и слабая прямая корреляция с психологическими симптомами (r=0,213, р=0,037), в то время как связи с сексуальными жалобами, которые являются наиболее значимыми симптомами гипогонадизма, установлено не было (р=0,479).

Кроме того, в нашем исследовании мы не определяли уровень ГСПГ и гонадотропинов, что планируется сделать на последующих этапах работы. Не определялся и уровень ТТГ и пролактина, при повышении которых также могут появляться лабораторные и клинические признаки гипогонадизма. Кроме того, в настоящем исследовании не проводилась оценка уровня эстрадиола, повышение которого также может сопровождать снижение уровня тестостерона, что важно в данном случае, учитывая возможное активирующее влияние провоспалительных цитокинов на ароматазу [14][15]. В дальнейших этапах исследования планируется определение вышеуказанных показателей.

## Направления дальнейших исследований

Одним из путей уточнения первичности взаимовлияния тестостерона и избытка массы тела при РА может быть проведение проспективного исследования с назначением тестостерон-заместительной терапии и динамической оценки индекса массы тела, объема талии, показателей углеводного и пуринового обменов, а также активности РА.

## Заключение

Отмечено отсутствие влияния уровня тестостерона и его дефицита на активность РА, однако пациенты со сниженным уровнем тестостерона имели лабораторные признаки нарушений углеводного (гипергликемия) и пуринового (гиперурикемия) обменов, а также повышение массы тела. Необходимы дальнейшие исследования с большей выборкой пациентов, а также проведение сравнительных исследований с другими ИВРЗ, оценивающих влияние тестостерон-заместительной терапии на метаболические показатели и активность РА.

## Дополнительная информация

Источники финансирования. Исследование выполнено в рамках государственного задания по теме №1021051503137-7 «Разработка персонализированной программы лечения рефрактерного ревматоидного артрита на основе изучения молекулярно-генетических и молекулярно-биологических предикторов. Создание и апробация регистра пациентов с ревматоидным артритом, резистентных к базисной противовоспалительной терапии».

Конфликт интересов. Авторы декларируют отсутствие явных и потенциальных конфликтов интересов, связанных с содержанием настоящей статьи.

Участие авторов. Паневин Т.С. — концепция и дизайн исследования, получение, интерпретация результатов, написание статьи; Роживанов Р.В. — дизайн исследования, анализ данных, интерпретация результатов, редактирование статьи; Зоткин Е.Г. — концепция исследования, написание и редактирование статьи; Диатроптов М.Е. — получение данных, редактирование статьи; Глухова С.И. — анализ данных, написание статьи; Самаркина Е.Ю. — получение данных, написание статьи. Все авторы одобрили финальную версию статьи перед публикацией, выразили согласие нести ответственность за все аспекты работы, подразумевающую надлежащее изучение и решение вопросов, связанных с точностью или добросовестностью любой части работы.
